# Numerical analysis of slip-enhanced flow over a curved surface with magnetized water-based hybrid nanofluid containing gyrotactic microorganisms

**DOI:** 10.1038/s41598-023-46214-9

**Published:** 2023-11-01

**Authors:** Humaira Yasmin, Showkat Ahmad Lone, Asifa Tassaddiq, Zehba Raizah, Hussam Alrabaiah, Anwar Saeed

**Affiliations:** 1 Department of Basic Sciences, Preparatory Year Deanship, King Faisal University, 31982 Al Ahsa, Saudi Arabia; 2 Department of Basic Sciences, College of Science and Theoretical Studies, Saudi Electronic University, 11673 Jeddah-M, Riyadh, Kingdom of Saudi Arabia; 3 Department of Basic Sciences and Humanities, College of Computer and Information Sciences, Majmaah University, 11952 Al-Majmaah, Saudi Arabia; 4 Department of Mathematics, College of Science, King Khalid University, Abha, Saudi Arabia; 5 College of Engineering, Al Ain University, Al Ain, United Arab Emirates; 6 Mathematics Department, Tafila Technical University, Tafila, Jordan; 7 Department of Mathematics, Abdul Wali Khan University, Mardan, 23200 Khyber Pakhtunkhwa Pakistan

**Keywords:** Engineering, Mathematics and computing

## Abstract

This article presents the two-dimensional flow of hybrid nanofluid comprising of gyrotactic microorganisms under the consequences of multiple slip conditions, magnetic field and thermal radiation across an elongating curved surface using porous media. The nanoparticles of TiO_2_ and Fe_3_O_4_ have dispersed in water for composition of hybrid nanofluid. Main equations of the problem are converted to ODEs by using an appropriate set of variables. Solution of the present model is determined with the help of bvp4c technique, which is explained in detail in the coming section. Validation of the current results is done versus the published work. The effects of various emerging factors on flow distributions have been considered and explained. Additionally, the slips conditions are incorporated to analyze various flow distributions. The present outcomes show that the rising magnetic factor lessens the velocity profile, whereas rises the temperature profile. The curvature factor has supported both temperature and velocity distributions. Growth in velocity, thermal, concentration, and microorganisms slip factors reduce the corresponding distributions. The greater impact of the embedded parameters is found on hybrid nanofluid flow when matched to nanofluid flow.

## Introduction

Nanofluid flow involves the movement of engineered mixtures comprising nanoparticles and a base fluid through channels or conduits. These nanoparticles, typically on the nanometer scale, are dispersed within the base fluid, altering its thermal and flow properties as established by Choi^1^. Nanofluids offer enhanced heat transfer capabilities due to increased thermal conductivity, while their flow behavior can be influenced by factors such as nanoparticle concentration, size, and shape^2^. Their applications range from electronics cooling to energy systems, though challenges related to stability, safety, and dispersion control continue to drive research in this multidisciplinary field^3^. Heat transfer in nanofluid flow is a phenomenon where the enhanced thermal conductance and altered flow features of nanofluids are harnessed to improve the efficiency of heat exchange processes^4^. Khan et al.^5^ inspected nanofluid flow through a porous conduit with impacts of microorganisms and noted that velocity panel declined and thermal panels escalated for higher porosity factor and nanoparticles’ concentration. Varun Kumar^6^ inspected a theoretical model for investigating nanoliquid flow on a stretched surface using influence of chemical reactivity and magnetic field. Acharya et al.^7^ studied nanofluid flow for growth in nano-layer and diameter of nanoparticles with thermal transference and have established 84.61% escalation in thermal transference for nano-layer. Shahid et al.^8^ examined computationally the experimental study for nanoparticles fluid flow on a permeable surface and have perceived that escalation in numerical factor like wall thickness, permeability, Forchheimer coefficient, and magnetic parameter has caused a deceleration in velocity. Hussain et al.^9^ discussed EMHD radiative nanoliquid flow on a stretched sheet. Gerdroodbary et al.^10^ used the computational algorithms to investigate the effect of non-uniform Kelvin force and variable MHD on the hydrothermal properties of a screw-type heat transfer device with the nanofluid flow. Their results demonstrate the skin friction of nanoliquid due to increasing screw measurement is significantly greater than the Reynolds number. Salahuddin^11^ has discussed several numerical methods in an inclusive way and has conveyed a combination of theories, MATLAB working exercises and examples. Awais and Salahuddin^12, 13^ investigated the flow, mass and heat propagation rates of a radiative non-Newtonian fluid on a parabolic sheet. The effect of thermal conductivity and radiation factors is discussed, and it is determined that skin friction increases with the magnetic flux. Ghazanfari et al.^14^ quantitatively investigated the effect of Al_2_O_3_ nanofluid and twisted tubes on the heating efficiency of heated tube exchangers and a shell. The analysis disclosed that using twisted tubes (rather than smooth tubes and pure water) and 20% nanofluid led to an 8% optimize in the heat transmission and a 40% reduction in pressure fall. Awais et al.^15, 16^ evaluated the non-Newtonian Casson nanoliquid flow across a slender parabola with variable viscosity, curvature factor and suction coefficient. Awais et al.^17^ discussed the mass and heat transmission through Eyring-Powell fluid in context of micro cantilever sensor and Darcy-Forchheimer medium, as well as the effects of thermal radiation and viscous dissipation. The findings indicated that the fluid velocity improves owing to the fluid coefficient and Hartman number, while the contrary impact is apparent due to the permeability and viscosity factor. Salahuddin et al.^18^ studied the energy and mass dissemination using the Cattaneo-Christov theory for the 2D Cross nanoliquid flow across the outermost layer of a parabola, under the influence of temperature-dependent viscosity. Manh et al.^19^ employed an analytical technique to illustrate the effects of nanomaterials on MHD flow within irregular plates and stated that the hydraulic boundary layer shrinks with rising values of Reynold number.

Hybrid nanofluid flow describes the movement of a mixture comprising two types of nanoparticles, each with distinct properties, dispersed within a base fluid through a conduit or channel or some other surface^20, 21^. These nanoparticles can be metallic, non-metallic, or carbon-based, and their combination aims to harness synergistic effects that enhance the fluid’s overall thermal, mechanical, and flow characteristics. Hybrid nanofluids offer the potential for even greater performance improvements in heat transfer, fluid dynamics, and other applications compared to single-component nanofluids^22^. However, achieving stable dispersion and understanding the complex interactions between different types of nanoparticles and the pure fluid is a challenge in this advanced area of research. Numerous researchers have diligently examined the augmented thermal transfer capabilities of hybrid nanofluids, delving into their potential to revolutionize heat transfer applications through the synergistic effects of combining distinct nanoparticles within a fluid medium^23, 24^. These investigations span experimental, theoretical, and computational approaches, striving to quantify improvements in thermal conductivity and convective heat transfer coefficients^25–27^. Gumber et al.^28^ examined thermal transference for nanoliquid flow on a surface using impacts of thermal radiations and have noted that Nusselt number is greater when considering the injective impact as opposed to the suction effect. Elattar et al.^29^ assessed the hybrid nanoparticles flow on a varying thickness stretched sheet and have noted that axial flow distribution has augmented with escalation in Hall current factor.

Fluid flow with chemical reactions is a complex field that involves the study of how fluids move and interact with each other while undergoing chemical reactions. This area of study is important in various scientific and engineering disciplines, including chemical engineering, environmental science, and materials science^30^. It encompasses both the fluid dynamics of the flow itself and the kinetics of the chemical reactions taking place within the flow. Thermal flow intertwined with chemical reactions constitutes a complex synergy where heat transfer mechanisms and chemical transformations intricately shape system dynamics^31^. The heat generated or absorbed during reactions affects the temperature gradients, which in turn influence reaction rates and equilibria. Such coupling is pivotal for understanding energy conversion, optimizing reaction yields, and ensuring process safety. Mathematical models and computational simulations enable insights into these complex processes, aiding in the design of advanced materials, improved reaction pathways, and innovative heat management strategies. Vaidya et al.^32^ inspected the collective effects chemical reactivity and varying thermal conductance on fluid flow in a conduit and discovered that velocity panel declined while temperature has escalated for higher magnetic factor. Kodi et al.^33^ debated on Casson MHD liquid flow on a permeable sheet using impacts of thermal diffusivity and chemical reactions. Biswas et al.^34^ treated computationally the Maxwell fluid flow using the impacts of chemical reactivity of first order and have noted that concentration panels retarded with upsurge in chemical reactivity factor. Reddy and Sreedevi^35^ inspected effects of double stratification and chemical reactivity on mass and thermal transportation and perceived that fluid concentration has affected by growth in reactivity factor. Patil et al.^36^ used MHD Prandtl fluid flow on a surface subjected to the control thermal radioactivity and chemical reactivity.

Joule heating, also identified as resistive heating, is a phenomenon that occurs when an electric current pass in a conductor, such as a wire or a resistor. It results in the conversion of electrical energy into heat energy due to the resistance encountered by the current as it flows through the material^37^. The heating effect is attributed to the collisions between the charged particles that constitute the electric current and the atoms or molecules of the conductor material. These collisions lead to the transfer of kinetic energy from the moving particles to the atoms or molecules, causing them to vibrate more vigorously. When fluid flow is involved in a system with Joule heating, the principles of Joule heating still apply, but now the heat generated due to the flow of electric current through the fluid can affect the behavior of the fluid itself^38^. This phenomenon is particularly relevant in systems where electrically conductive fluids, such as electrolytes or certain types of liquid metals, are present. Rafique et al.^39^ observed the upshot of various nanoparticles shapes and nonlinear velocity for MHD fluid flow on stretched surface by employing Joule heating and distinguished that velocity panel has dropped and thermal panels have escalated with progress in magnetic factor. Abo-Dahab et al.^40^ deliberated on double diffusion MHD fluid flow by means of thermal radiations, Joule heating and thermal absorption/generation effects. Rasool et al.^41^ treated computationally the nanoliquid flow on a sheet and obtained double solutions to their problem. Irfan et al.^42^ calculated thermally the performance of radiative and mixed convective fluid flow and have noted that Nusselt number has escalated by 3.69% and declined by 7.75% for respective escalation in thermophoresis factor and Eckert number. Khan et al.^43^ scrutinized the irreversibility creation for thermally analyzed MHD fluid flow on a gyrating cylinder using Joule heating effects.

Gyrotactic microorganisms are the organisms, like bacteria and algae that exhibit gyrotaxis which is a unique behavior in response to gravity and fluid flow gradients^44^. In thermal analysis with gyrotactic microorganisms within fluid flow, the interactions between heat, fluid motion, and gyrotaxis are studied. Gyrotactic microorganisms, responding to gravity and fluid flow gradients, influence the distribution and movement of microorganisms within the fluid^45^. As the microorganisms position themselves at specific depths in response to varying temperature and nutrient conditions, they can modify the local thermal gradients by altering the fluid mixing patterns through their helical swimming paths^46^. Consequently, the temperature distribution within the fluid becomes non-uniform due to the collective effects of fluid flow-induced heat transference and the microorganisms’ gyrotactic responses. Kada et al.^47^ inspected the significance of microbes on bio-convective investigation for Williamson radiative fluid flow. Anjum et al.^48^ debated on bio-convective and radiative fluid flow using chemical reactivity and microorganisms effects and have noted that density of microorganisms has retarded with escalation in Peclet number. Fatima et al.^49^ studied the fluid flow with the impacts of microbes. Shahzad et al.^50^ discussed bio-convective nanofluid flow through disks using impacts of Darcy-Forchheimer model and noted that microorganisms’ distribution has de-escalated with upsurge in Peclet and bio-convective Lewis numbers.

Based on the studied literature, it has found that very less work has done on the water-based hybrid nanofluid flow comprising of gyrotactic microorganisms under the consequences of multiple slip conditions, across a stretching curved surface using porous media which is considered in this work. The nanoparticles of TiO_2_ and Fe_3_O_4_ have dispersed in water for composition of hybrid nanofluid. Various effects like the impacts of magnetic fields, thermal radiation, porous media, Joule heating, thermophoresis and Brownian motion are all accounted for in the analysis. Additionally, the slips conditions are incorporated to analyze all flow distributions in detail. The work is formulated in Section "Formulation of problem" with its computational analysis in Section "Numerical solution". Validation of the present results is established with available data in Section "Validation". The Results of current work has discussed in Section "Discussion of results" and has concluded in Section "Conclusions".

## Formulation of problem

**Figure 1:**
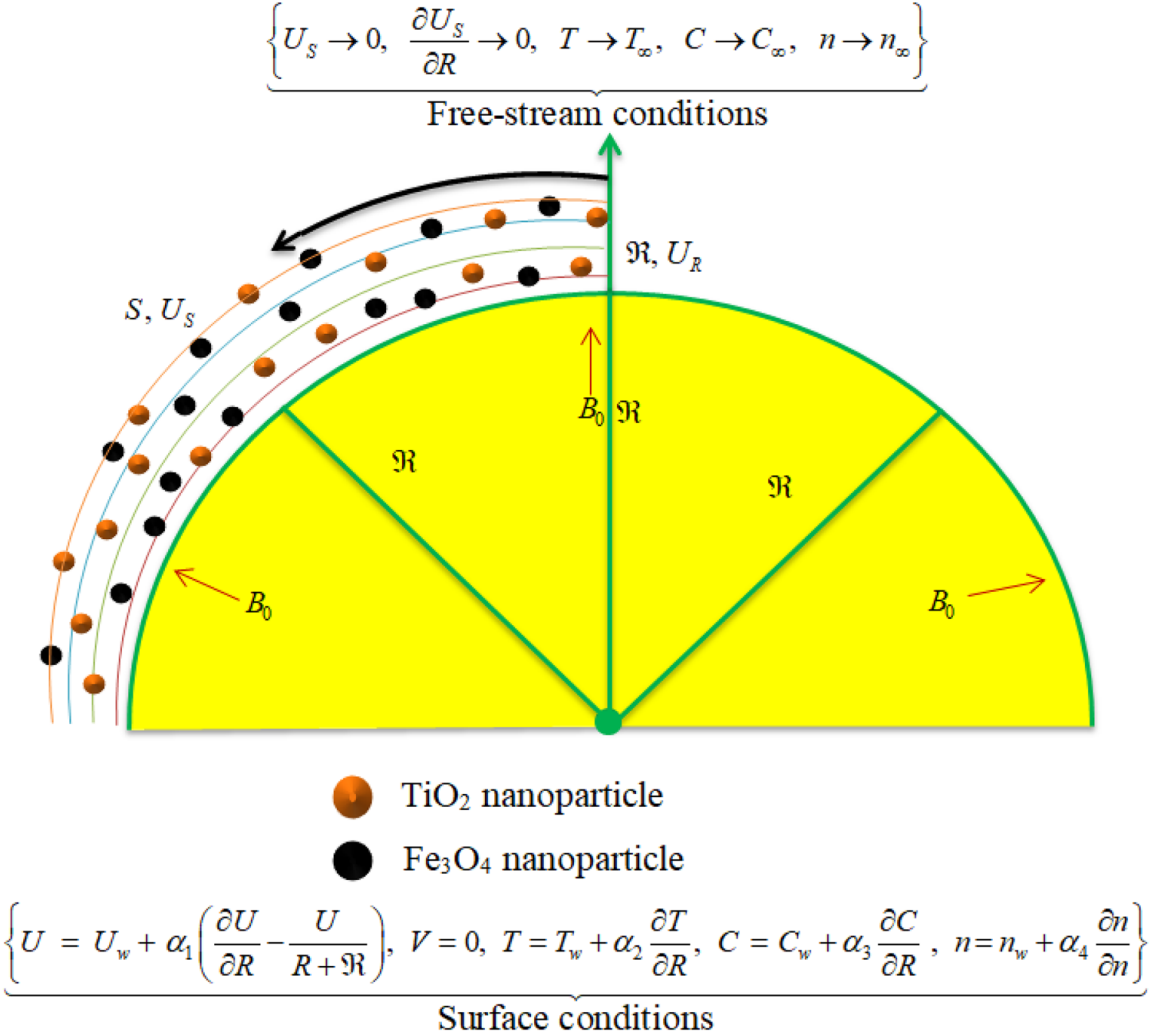
Physical illustration of the flow problem.

Take 2D flow of hybrid nanoliquid comprising of gyrotactic microorganisms on an elongating curved surface using porous media. The nanoparticles of *TiO*_*2*_ and Fe_3_O_4_ have dispersed in water for composition of hybrid nanofluid. A curvilinear coordinate system ($R,S$) having $U$ and $V$ as velocities in $S -$ and $R -$ directions are considered. The surface stretches with velocity $U_{w} = aS$, where $a > 0$ is constant, along $S -$ direction while $R$ is the normal direction to the fluid flow (see Fig. 1). The temperature, nanoparticle concentration, and concentration of microorganisms are denoted respectively by $T$, $C$ and $n$ with corresponding values at the surface and free sream are $T_{w}$, $C_{w}$ and $n_{w}$, and $T_{\infty }$, $C_{\infty }$ and $n_{\infty }$. $B_{0}$ is strength of magnetic field acted in normal direction to fluid flow. Additionally, the slips conditions are incorporated to analyze the fluid flow.

The main equations take the form^51–55^:

1 $$\frac{\partial V}{{\partial R}} + \left( {\frac{\Re }{\Re + R}} \right)\frac{\partial U}{{\partial S}} + \left( {\frac{V}{\Re + R}} \right)\, = 0,$$

2 $$\left( {\frac{{U^{2} }}{R + \Re }} \right) = - \frac{1}{{\rho_{hnf} }}\frac{\partial p}{{\partial R}},$$

3 $$\begin{gathered} V\frac{\partial U}{{\partial R}} + \left( {\frac{\Re }{\Re + R}} \right)U\frac{\partial U}{{\partial S}} + \left( {\frac{1}{\Re + R}} \right)UV = - \frac{1}{{\rho_{hnf} }}\left( {\frac{\Re }{\Re + R}} \right)\frac{\partial p}{{\partial S}} + \hfill \\ \frac{{\mu_{hnf} }}{{\rho_{hnf} }}\left( {\frac{{\partial^{2} U}}{{\partial R^{2} }} + \frac{\partial U}{{\partial R}}\left( {\frac{1}{\Re + R}} \right) - U\left( {\frac{1}{\Re + R}} \right)^{2} } \right) - \frac{{\sigma_{hnf} }}{{\rho_{hnf} }}B_{0}^{2} U - \frac{{\nu_{hnf} }}{{k_{p} }}U, \hfill \\ \end{gathered}$$

4 $$\begin{gathered} V\frac{\partial T}{{\partial R}} + \left( {\Re + R} \right)U\frac{\partial T}{{\partial S}} = \frac{{k_{hnf} }}{{\left( {\rho C_{p} } \right)_{hnf} }}\left[ {\left( {\frac{1}{\Re + R}} \right)\frac{\partial T}{{\partial R}} + \frac{{\partial^{2} T}}{{\partial R^{2} }}} \right] + \frac{{\sigma_{hnf} }}{{\left( {\rho C_{p} } \right)_{hnf} }}B_{0}^{2} U^{2} \hfill \\ + \frac{{\left( {\rho C_{p} } \right)_{np} }}{{\left( {\rho C_{p} } \right)_{hnf} }}\left( {D_{B} \frac{\partial T}{{\partial R}}\frac{\partial C}{{\partial R}} + \frac{{D_{T} }}{{T_{\infty } }}\left( {\frac{\partial T}{{\partial R}}} \right)^{2} } \right) - \frac{1}{{\left( {\rho C_{p} } \right)_{Thnf} \left( {\Re + R} \right)}}\frac{\partial }{\partial R}\left( {\Re + R} \right)q_{r} , \hfill \\ \end{gathered}$$

5 $$v\frac{\partial C}{{\partial R}} + \frac{\partial C}{{\partial S}}\left( {\frac{\Re U}{{\Re + R}}} \right) = D_{B} \left[ {\frac{\partial C}{{\partial R}}\left( {\frac{1}{\Re + R}} \right) + \frac{{\partial^{2} C}}{{\partial R^{2} }}} \right] + \frac{{D_{T} }}{{T_{\infty } }}\left( {\frac{{\partial^{2} T}}{{\partial R^{2} }} + \left( {\frac{1}{\Re + R}} \right)\frac{\partial T}{{\partial R}}} \right),$$

6 $$V\frac{\partial n}{{\partial R}} + \left( {\frac{U\Re }{{R + \Re }}} \right)\frac{\partial n}{{\partial S}} = D_{m} \left[ {\frac{\partial n}{{\partial R}}\left( {\frac{1}{R + \Re }} \right) + \frac{{\partial^{2} n}}{{\partial R^{2} }}} \right] + \frac{{bW_{c} }}{{\left( {C_{w} - C_{\infty } } \right)}}\left( {\frac{\partial }{\partial R}\left( {n\frac{\partial C}{{\partial R}}} \right)} \right),$$

with boundary conditions:

7 $$\left\{ \begin{gathered} U\,\,\, = \,U_{w} \, + \,\,\alpha_{1} \left( {\frac{\partial U}{{\partial R}} - \frac{U}{\Re + R}} \right),\,\,\,V = 0,\,\,\,T = T_{w} + \alpha_{2} \frac{\partial T}{{\partial R}},\,\,\,C\,\, = \,\,C_{w} + \alpha_{3} \frac{\partial C}{{\partial R}}\,\,,\,\,\,n\, = n_{w} + \alpha_{4} \frac{\partial n}{{\partial n}}\,\,\,{\text{at}}\,\,\,R = 0, \hfill \\ \,\,\,\,\,\,\,\,\,\,\,\,\,\,\,\,\,\,\,\,\,\,\,\,\,\,\,\,\,\,\,\,U \to 0,\,\,\frac{\partial U}{{\partial R}} \to 0,\,\,\,\,T \to T_{\infty } ,\,\,\,C \to C_{\infty } ,\,\,\,n \to n_{\infty } \,\,\,{\text{when}}\,\,\,R \to \infty \hfill \\ \end{gathered} \right\}$$

Above $q_{r}$ (radiative heat flux) is described as:

8 $$q_{r} = \frac{{4\sigma^{*} \,\partial T^{4} }}{{3k^{*} \,\,\partial R}} = \frac{{16\sigma^{*} T_{\infty }^{3} }}{{3k^{*} }}\frac{\partial T}{{\partial R}}.$$

The thermophysical correlations of the hybrid nanofluid are defined as:

9 $$\left\{ \begin{gathered} \mu_{hnf} = \frac{{\mu_{f} }}{{\left( {1 - \psi_{1} } \right)^{2.5} \left( {1 - \psi_{2} } \right)^{2.5} }},\,\,\,\frac{{\rho_{hnf} }}{{\rho_{f} }} = \left( {1 - \psi_{2} } \right)\left\{ {\left( {1 - \psi_{1} } \right) + \psi_{1} \frac{{\rho_{{P_{1} }} }}{{\rho_{f} }}} \right\} + \psi_{2} \frac{{\rho_{{P_{2} }} }}{{\rho_{f} }}, \hfill \\ \frac{{\left( {\rho C_{p} } \right)_{hnf} }}{{\left( {\rho C_{p} } \right)_{f} }} = \left( {1 - \psi_{2} } \right)\left\{ {\left( {1 - \psi_{1} } \right) + \psi_{1} \frac{{\left( {\rho C_{p} } \right)_{{P_{1} }} }}{{\left( {\rho C_{p} } \right)_{f} }}} \right\} + \psi_{2} \frac{{\left( {\rho C_{p} } \right)_{{P_{2} }} }}{{\left( {\rho C_{p} } \right)_{f} }}, \hfill \\ \frac{{k_{hnf} }}{{k_{nf} }} = \frac{{k_{{P_{2} }} + 2k_{nf} - 2\left( {k_{nf} - k_{{P_{2} }} } \right)\psi_{2} }}{{k_{{P_{2} }} + 2k_{nf} + \left( {k_{nf} - k_{{P_{2} }} } \right)\psi_{2} }},\,\,\,\,\frac{{k_{nf} }}{{k_{f} }} = \frac{{k_{{P_{1} }} + 2k_{f} - 2\left( {k_{f} - k_{{P_{1} }} } \right)\psi_{1} }}{{k_{{p_{1} }} + 2k_{f} + \left( {k_{f} - k_{{P_{1} }} } \right)\psi_{1} }}, \hfill \\ \frac{{\sigma_{hnf} }}{{\sigma_{nf} }} = \frac{{\left( {1 + 2\psi_{2} } \right)\sigma_{{P_{2} }} + \left( {1 - 2\psi_{2} } \right)\sigma_{nf} }}{{\left( {1 - \varphi_{2} } \right)\sigma_{{p_{2} }} + \left( {1 + \psi_{2} } \right)\sigma_{f} }},\,\,\,\,\frac{{\sigma_{nf} }}{{\sigma_{f} }} = \frac{{\left( {1 + 2\psi_{1} } \right)\sigma_{{P_{1} }} + \left( {1 - 2\psi_{1} } \right)\sigma_{f} }}{{\left( {1 - \psi_{1} } \right)\sigma_{{P_{1} }} + \left( {1 + \psi_{1} } \right)\sigma_{f} }}. \hfill \\ \end{gathered} \right\}$$

The experimental values of the thermophysical characteristics are defined in Table 1.

**Table 1:** **T**hermophysical properties for nanoparticles and base fluid.

Properties	${\text{H}}_{2} {\text{O}}$	${\text{TiO}}_{2}$	${\text{Fe}}_{3} {\text{O}}_{4}$
$\rho$	997.1	4250	5180
$C_{p}$	4179	686.2	670
$k$	0.613	8.9538	9.7
$\sigma$	$5.50\,\times\,10^{ - 6}$	$6.27\,\times\,10^{ - 5}$	25,000

The set of variables used for transformation is described as:

10 $$\left\{ \begin{gathered} \xi = \sqrt {\frac{a}{{\nu_{f} }}} R,\,\,\,\,\,U = U_{w} f^{\prime}\left( \eta \right),\,\,\,\,V = \frac{R + \Re }{\Re }\sqrt {a\nu_{f} } f\left( \xi \right),\,\,\,\, \hfill \\ \phi \left( \xi \right) = \frac{{C - C_{\infty } }}{{C_{w} - C_{\infty } }},\,\,\,\,\theta \left( \xi \right) = \frac{{T - T_{\infty } }}{{T_{w} - T_{\infty } }},\,\,\,\chi \left( \xi \right) = \frac{{n - n_{\infty } }}{{n_{w} - n_{\infty } }},\,\,\,p = \rho_{f} a^{2} S^{2} P\left( \xi \right). \hfill \\ \end{gathered} \right\}$$

Using (10), we have from above:

11 $$P^{\prime } \left( \xi \right) = \frac{{f^{\prime 2} \left( \xi \right)}}{\xi + \Omega },$$

12 $$\begin{gathered} \left( {\frac{{E_{1} }}{{E_{2} }}} \right)\left( {f^{\prime\prime\prime}\left( \xi \right) + \left( {\frac{1}{\xi + \Omega }} \right)f^{\prime\prime}\left( \xi \right) - \left( {\frac{1}{{\left( {\xi + \Omega } \right)^{2} }}} \right)f^{\prime}\left( \xi \right)} \right) - \left( {\frac{{E_{3} }}{{E_{2} }}} \right)Mf^{\prime}\left( \xi \right) + \left( {\frac{\Omega }{{\left( {\xi + \Omega } \right)}}} \right)f\left( \xi \right)f^{\prime\prime}\left( \xi \right) \hfill \\ \,\,\,\,\, - \left( {\frac{\Omega }{{\left( {\xi + \Omega } \right)}}} \right)f^{{\prime}{2}} \left( \xi \right) + \left( {\frac{\Omega }{{\left( {\xi + \Omega } \right)^{2} }}} \right)f^{\prime}\left( \xi \right)f\left( \xi \right) - \left( {\frac{2}{{E_{2} }}} \right)\left( {\frac{\Omega }{{\left( {\xi + \Omega } \right)}}} \right)P\left( \xi \right) - \left( {\frac{{E_{1} }}{{E_{2} }}} \right)\lambda f^{\prime}\left( \xi \right) = 0, \hfill \\ \end{gathered}$$

13 $$\begin{gathered} P\left( \xi \right) = E_{1} \left( {\left( {\frac{\xi + \Omega }{{2\Omega }}} \right)f^{\prime\prime\prime}\left( \xi \right) + \left( {\frac{1}{2\Omega }} \right)f^{\prime\prime}\left( \xi \right) - \left( {\frac{1}{{2\Omega \left( {\Omega + \xi } \right)}}} \right)f^{\prime}\left( \xi \right)} \right) - E_{3} \frac{{\left( {\xi + \Omega } \right)}}{2\Omega }Mf^{\prime}\left( \xi \right) \hfill \\ \,\,\, + E_{2} \left( {\frac{1}{2}f\left( \xi \right)f^{\prime\prime}\left( \xi \right) - \frac{1}{2}f^{{\prime}{2}} \left( \xi \right) + \left( {\frac{1}{{2\left( {\Omega + \xi } \right)}}} \right)f\left( \xi \right)f^{\prime}\left( \xi \right)} \right) - E_{1} \frac{{\left( {\xi + \Omega } \right)}}{2\Omega }\lambda f^{\prime}\left( \xi \right), \hfill \\ \end{gathered}$$

Differentiating Eq. (13) w.r.t $\xi$, we have:

14 $$\begin{gathered} E_{1} \left( {f^{{\left( {iv} \right)}} \left( \xi \right) + \left( {\frac{2}{{\left( {\xi + \Omega } \right)}}} \right)f^{\prime\prime\prime}\left( \xi \right) - \left( {\frac{1}{{\left( {\Omega + \xi } \right)^{2} }}} \right)f^{\prime\prime}\left( \xi \right) + \left( {\frac{1}{{\left( {\Omega + \xi } \right)^{3} }}} \right)f^{\prime}\left( \xi \right)} \right) - E_{3} M\left( {f^{\prime\prime}\left( \xi \right) + \left( {\frac{1}{{\left( {\xi + \Omega } \right)}}} \right)f^{\prime}\left( \xi \right)} \right) \hfill \\ \,\,\,\,\,\,\, + E_{2} \left( \begin{gathered} \left( {\frac{\Omega }{{\left( {\Omega + \xi } \right)}}} \right)\left\{ {f\left( \xi \right)f^{\prime\prime\prime}\left( \xi \right) - f^{\prime\prime}\left( \xi \right)f^{\prime}\left( \xi \right)} \right\} + \frac{\Omega }{{\left( {\Omega + \xi } \right)^{2} }} \hfill \\ \times \left\{ {f\left( \xi \right)f^{\prime\prime}\left( \xi \right) - f^{{\prime}{2}} \left( \xi \right)} \right\} - \left( {\frac{\Omega }{{\left( {\Omega + \xi } \right)^{3} }}} \right)f\left( \xi \right)f^{\prime}\left( \xi \right) \hfill \\ \end{gathered} \right) - E_{1} \lambda \left( {f^{\prime\prime}\left( \xi \right) + f^{\prime}\left( \xi \right)\left( {\frac{1}{{\left( {\xi + \Omega } \right)}}} \right)} \right) = 0, \hfill \\ \end{gathered}$$

15 $$\begin{gathered} \frac{{E_{4} }}{{E_{5} }}\frac{1}{\Pr }\left( {1 - \frac{4}{3}\frac{{R_{d} }}{{E_{5} }}} \right)\left( {\theta^{\prime\prime}\left( \xi \right) + \frac{1}{\xi + \Omega }\theta^{\prime}\left( \xi \right)} \right) + \frac{\Omega }{\xi + \Omega }f\left( \xi \right)\theta^{\prime}\left( \xi \right) \hfill \\ \,\,\,\,\, + \frac{1}{{E_{5} }}\left( {N_{b} \theta^{\prime}\left( \xi \right)\phi^{\prime}\left( \xi \right) + N_{t} \theta^{{\prime}{2}} \left( \xi \right)} \right) + \frac{{E_{3} }}{{E_{5} }}MEcf^{{\prime}{2}} \left( \xi \right) = 0, \hfill \\ \end{gathered}$$

16 $$\phi^{\prime\prime}\left( \xi \right) + \frac{1}{\xi + \Omega }\phi^{\prime}\left( \xi \right) + \frac{{N_{t} }}{{N_{b} }}\left( {\theta^{\prime\prime}\left( \xi \right) + \frac{1}{\xi + \Omega }\theta^{\prime}\left( \xi \right)} \right) + \frac{\Omega }{\xi + \Omega }S_{c} f\left( \xi \right)\phi^{\prime}\left( \xi \right) = 0,$$

17 $$\chi^{\prime\prime}\left( \xi \right) + \frac{1}{\xi + \Omega }\chi^{\prime}\left( \xi \right) + P_{b} \left( {\chi^{\prime}\left( \xi \right)\phi^{\prime} + \left( {\delta + \chi \left( \xi \right)} \right)\phi^{\prime\prime}\left( \xi \right)} \right) + \frac{\Omega }{\xi + \Omega }S_{b} \,f\left( \xi \right)\chi^{\prime}\left( \xi \right) = 0,$$

Related constraints at boundaries are:

18 $$\left\{ \begin{aligned} & f\left( 0 \right) = 0,\,\,f^{\prime}\left( 0 \right) = 1 - \gamma_{1} \left( {\frac{{f^{\prime}\left( 0 \right)}}{{\left( {\xi + \Omega } \right)}} - f^{\prime\prime}\left( 0 \right)} \right),\,\,\theta \left( 0 \right) = 1 + \gamma_{2} \theta^{\prime}\left( 0 \right),\,\,\,\phi = 1 + \gamma_{3} \,\phi^{\prime}\left( 0 \right),\,\chi = 1 + \gamma_{4} \chi^{\prime}\left( 0 \right), \\ & f^{\prime}\left( 0 \right) \to 0,\,\,\,\,f^{\prime\prime}\left( 0 \right) \to 0,\,\,\,\,\theta \left( 0 \right) \to 0,\,\,\,\,\phi \left( 0 \right) \to 0,\,\,\,\,\chi \left( 0 \right) \to 0. \\ \end{aligned} \right\}\,$$

Above, $\Pr \left( { = \frac{{\nu_{f} \left( {\rho C_{p} } \right)_{f} }}{{k_{f} }}} \right)$ is Prandtl number, $Nb\left( { = \frac{{\left( {\rho C_{p} } \right)_{np} D_{B} \left( {C_{w} - C_{\infty } } \right)}}{{\left( {\rho C_{p} } \right)_{f} \nu_{f} }}} \right)$ is Brownian motion factor, $Nt\left( { = \frac{{\left( {\rho C_{p} } \right)_{np} D_{T} \left( {T_{w} - T_{\infty } } \right)}}{{\left( {\rho C_{p} } \right)_{f} \nu_{f} T_{\infty } }}} \right)$ is thermophoretic factor, $M\left( { = \frac{{\sigma_{f} B_{0}^{2} }}{{\rho_{f} a}}} \right)$ is magnetic factor, $Ec\left( { = \frac{{\left( {U_{w} } \right)^{2} }}{{\left( {C_{p} } \right)_{f} \left( {T_{w} - T_{\infty } } \right)}}} \right)$ is Eckert number, $P_{b} = \left( {\,\frac{{bW_{c} }}{{D_{m} }}} \right)$ is bioconvection Peclet number, $Sc\left( { = \frac{{\nu_{f} }}{{D_{B} }}} \right)$ is the Schmidt number, $\delta = \frac{{n_{\infty } }}{{n_{w} - n_{\infty } }}$ is concentration difference factor, $R_{d} = \frac{{4\sigma^{*} T_{\infty }^{3} }}{{k_{f} k^{*} }}$ is thermal radiation factor, $\lambda = \frac{{\mu_{f} }}{{\rho_{f} ak_{p} }}$ is porosity factor, $\Omega = \Re \sqrt {\frac{a}{{\nu_{f} }}} R$ is curvature factor and is bioconvection Lewis number $S_{b} = \frac{{\nu_{f} }}{{D_{m} }}$.

The interested quantities are described as:

19 $$C_{fS} = \frac{{\tau_{RS} }}{{\left( {U_{w} } \right)^{2} }},\,\,\,\,Nu_{S} = \frac{{Sq_{S} }}{{k_{f} \left( {T_{w} - T_{\infty } } \right)}},\,\,\,\,Sh_{S} = \frac{{Sq_{j} }}{{D_{B} \left( {C_{w} - C_{\infty } } \right)}},\,\,\,\,Mn_{S} = \frac{{Sq_{m} }}{{D_{m} \left( {n_{w} - n_{\infty } } \right)}},$$

where

20 $$\,\tau_{RS} = \mu_{hnf} \left. {\left( {\frac{\partial U}{{\partial R}} - \frac{U}{R + \Re }} \right)} \right|_{R = 0} ,\,\,\,\,\,\,q_{S} = - k_{hnf} \left. {\frac{\partial T}{{\partial R}}} \right|_{R = 0} ,\,\,\,\,\,q_{j} = - D_{B} \left. {\frac{\partial C}{{\partial R}}} \right|_{R = 0} ,\,\,\,\,q_{m} = - D_{m} \left. {\frac{\partial N}{{\partial R}}} \right|_{R = 0} .$$

Using the similarity variables defined above, we have:

21 $$\left\{ \begin{gathered} {\text{Re}}_{S}^{1/2} C_{fS} \, = \frac{{\mu_{hnf} }}{{\mu_{f} }}\left( {f^{\prime\prime}\left( {\xi = 0} \right) - \frac{1}{\Omega }\,f^{\prime}\left( {\xi = 0} \right)} \right), \hfill \\ \,\,\,\,\,\,\,\,\,\,\,\,\,{\text{Re}}_{S}^{ - 1/2} {\text{Nu}}_{S} = - \frac{{k_{hnf} }}{{k_{f} }}\theta^{\prime}\left( {\xi = 0} \right),\,\,\,\, \hfill \\ \,\,\,\,\,\,\,\,\,\,\,\,\,\,\,\,\,\,{\text{Re}}_{S}^{ - 1/2} Sh_{S} = - \phi^{\prime}\left( {\xi = 0} \right),\,\,\,\, \hfill \\ \,\,\,\,\,\,\,\,\,\,\,\,\,\,\,\,{\text{Re}}_{S}^{ - 1/2} Mn_{S} = - \chi^{\prime}\left( {\xi = 0} \right). \hfill \\ \end{gathered} \right\}$$

${\text{Re}}_{S} = \frac{{aS^{2} }}{{\nu_{f} }}$ is (local) Reynolds number.

## Numerical solution

The numerical solutions of well-known ODEs that represent the flow, energy transport and mass transport Eqs. (14–17) and boundary conditions in Eq. (18) is discussed in this section. The numerical results are obtained using the bvp4c built-in MATLAB technique. A finite difference method known as the bvp4c makes use of the three-stage Lobatto III formula. This formula uses collocation, and the collocation polynomial offers an accurate fourth-order *C*^*1*^-continuous solution for the specified interval. The residual of the solution serves as a strong foundation for the mesh and error control. To determine the numerical solution of the suggested model by using bvp4c scheme, we transform nonlinear differential equations of higher order to linear equation of first order. Therefore, let us assume that:

22 $$\left\{ \begin{gathered} \,\,\,f^{\prime}\left( \xi \right) = \nabla \left( 2 \right),\,\,\,f\left( \xi \right) = \nabla \left( 1 \right),\,\,\,\,\,f^{\prime\prime\prime}\left( \xi \right) = \nabla \left( 4 \right),\,\,f^{\prime\prime}\left( \xi \right) = \nabla \left( 3 \right),\,\,\,\,f^{(iv)} \left( \xi \right) = \nabla^{\prime}\left( 4 \right), \hfill \\ \,\,\,\,\,\,\,\,\,\,\,\,\,\,\,\,\,\,\,\,\,\,\,\,\,\,\,\,\,\,\,\,\,\,\,\,\theta \left( \xi \right) = \nabla \left( 5 \right),\,\,\,\,\theta^{\prime}\left( \xi \right) = \nabla \left( 6 \right),\,\,\,\,\theta^{\prime\prime}\left( \xi \right) = \nabla^{\prime}\left( 6 \right), \hfill \\ \,\,\,\,\,\,\,\,\,\,\,\,\,\,\,\,\,\,\,\,\,\,\,\,\,\,\,\,\,\,\,\,\,\,\,\,\phi \left( \xi \right) = \nabla \left( 7 \right),\,\,\,\,\phi^{\prime}\left( \xi \right) = \nabla \left( 8 \right),\,\,\,\,\phi^{\prime\prime}\left( \xi \right) = \nabla^{\prime}\left( 8 \right), \hfill \\ \,\,\,\,\,\,\,\,\,\,\,\,\,\,\,\,\,\,\,\,\,\,\,\,\,\,\,\,\,\,\,\,\chi \left( \xi \right) = \nabla \left( 9 \right),\,\,\,\,\chi^{\prime}\left( \xi \right) = \nabla \left( {10} \right),\,\,\,\,\chi^{\prime\prime}\left( \xi \right) = \nabla^{\prime}\left( {10} \right). \hfill \\ \end{gathered} \right\}$$

Using these transformations, the set of ODEs can be outlined as:

23 $$\nabla^{\prime}\left( 4 \right) = - \frac{{\left\{ \begin{gathered} E_{1} \left( {\frac{2}{{\left( {\xi + \Omega } \right)}}\nabla \left( 4 \right) - \frac{1}{{\left( {\xi + \Omega } \right)^{2} }}\nabla \left( 3 \right) + \frac{1}{{\left( {\xi + \Omega } \right)^{3} }}\nabla \left( 2 \right)} \right) - E_{3} M\left( {\nabla \left( 3 \right) + \frac{1}{{\left( {\xi + \Omega } \right)}}\nabla \left( 2 \right)} \right) \hfill \\ + E_{2} \left( \begin{gathered} \frac{\Omega }{{\left( {\xi + \Omega } \right)}}\left\{ {\nabla \left( 1 \right)\nabla \left( 4 \right) - \nabla \left( 3 \right)\nabla \left( 2 \right)} \right\} + \frac{\Omega }{{\left( {\xi + \Omega } \right)^{2} }} \hfill \\ \times \left\{ {\nabla \left( 1 \right)\nabla \left( 3 \right) - \left( {\nabla \left( 2 \right)} \right)^{2} } \right\} - \frac{\Omega }{{\left( {\xi + \Omega } \right)^{3} }}\nabla \left( 1 \right)\nabla \left( 2 \right) \hfill \\ \end{gathered} \right) - E_{1} \lambda \left( {\nabla \left( 3 \right) + \frac{1}{{\left( {\xi + \Omega } \right)}}\nabla \left( 2 \right)} \right) \hfill \\ \end{gathered} \right\}}}{{E_{1} }},$$

24 $$\nabla^{\prime}\left( 6 \right) = - \frac{{\left\{ \begin{gathered} \,\frac{{E_{4} }}{{E_{5} }}\frac{1}{\Pr }\left( {1 - \frac{4}{3}\frac{{R_{d} }}{{E_{5} }}} \right)\left( { + \frac{1}{\xi + \Omega }\nabla \left( 6 \right)} \right) + \frac{\Omega }{\xi + \Omega }\nabla \left( 1 \right)\nabla \left( 6 \right) + \hfill \\ \frac{1}{{E_{5} }}\left( {N_{b} \nabla \left( 6 \right)\nabla \left( 8 \right) + N_{t} \left( {\nabla \left( 6 \right)} \right)^{2} } \right) + \frac{{E_{3} }}{{E_{5} }}MEc\left( {\nabla \left( 2 \right)} \right)^{2} \left( \xi \right) \hfill \\ \end{gathered} \right\}}}{{\frac{{E_{4} }}{{E_{5} }}\frac{1}{\Pr }\left( {1 - \frac{4}{3}\frac{{R_{d} }}{{E_{5} }}} \right)}},$$

25 $$\nabla^{\prime}\left( 8 \right) = - \left\{ {\frac{1}{\xi + \Omega }\nabla \left( 8 \right) + \frac{{N_{t} }}{{N_{b} }}\left( {\nabla^{\prime}\left( 6 \right) + \frac{1}{\xi + \Omega }\nabla \left( 6 \right)} \right) + \frac{\Omega }{\xi + \Omega }S_{c} \nabla \left( 1 \right)\nabla \left( 8 \right)} \right\},$$

26 $$\nabla^{\prime}\left( {10} \right) = - \left\{ {\frac{1}{\xi + \Omega }\nabla \left( {10} \right) + P_{b} \left( {\nabla \left( {10} \right)\nabla \left( 8 \right) + \left( {\delta + \nabla \left( 9 \right)} \right)\nabla^{\prime}\left( 8 \right)} \right) + \frac{\Omega }{\xi + \Omega }S_{b} \,\nabla \left( 2 \right)\nabla \left( {10} \right)} \right\},$$

with boundary conditions:

27 $$\left\{ \begin{gathered} \nabla_{a} \left( 1 \right) - 0,\,\,\nabla_{a} \left( 2 \right) - 1 + \gamma_{1} \left( {\frac{{\nabla_{a} \left( 2 \right)}}{{\left( {\xi + \Omega } \right)}} - \nabla_{a} \left( 3 \right)} \right), \hfill \\ \,\,\,\,\,\,\,\,\,\,\,\,\,\,\,\,\,\,\,\,\nabla_{b} \left( 2 \right) - 0,\,\,\,\,\nabla_{b} \left( 3 \right) - 0, \hfill \\ \,\,\,\,\,\,\,\,\,\,\,\,\,\,\,\,\,\,\,\,\,\nabla_{a} \left( 5 \right) - 1 - \gamma_{2} \nabla_{a} \left( 6 \right),\,\,\, \hfill \\ \,\,\,\,\,\,\,\,\,\,\,\,\,\,\,\,\,\,\,\,\,\,\,\,\,\,\,\,\,\,\nabla_{b} \left( 5 \right) - 0 \hfill \\ \,\,\,\,\,\,\,\,\,\,\,\,\,\,\,\,\,\,\,\,\,\nabla_{a} \left( 7 \right) - 1 - \gamma_{3} \nabla_{a} \left( 8 \right),\, \hfill \\ \,\,\,\,\,\,\,\,\,\,\,\,\,\,\,\,\,\,\,\,\,\,\,\,\,\,\,\,\,\,\nabla_{b} \left( 7 \right) - 0 \hfill \\ \,\,\,\,\,\,\,\,\,\,\,\,\,\,\,\,\,\,\,\,\,\nabla_{a} \left( 9 \right) - 1 - \gamma_{4} \nabla \left( {10} \right), \hfill \\ \,\,\,\,\,\,\,\,\,\,\,\,\,\,\,\,\,\,\,\,\,\,\,\,\,\,\,\,\,\,\nabla_{b} \left( 9 \right) - 0. \hfill \\ \end{gathered} \right\}\,$$

Here, the subscripts $a$ and $b$ shows the initial and boundary conditions, respectively. This method has the following benefits:

1. Within minimal effort, we can solve highly nonlinear systems.
2. By using this method, we are free to set the tolerance for error (the tolerance for error for the present case is 1 × 10^–6^).
3. We can solve those challenging problems which cannot be solved by using analytical methods.
4. The solution of this technique is much easier and faster than other techniques.

## Validation

In order to validate the applied technique with the formally available results of different researches, Table 2 and 3 are shown. Table 2 shows comparison of the ${\text{Re}}_{S}^{1/2} C_{fS}$ for variations in $\Omega$ and $\psi_{1} = \psi_{2} = \lambda = M = 0$. Table 3 shows the comparison of the ${\text{Re}}_{S}^{ - 1/2} {\text{Nu}}_{S}$ for variations in $\Pr$ when $\Omega \to \infty$ and rest of factors are fixed as zero. From both the Tables, we confirmed that the results of the present model are very close to those of the published results. Thus, the present model and the applied numerical technique are both applicable.

**Table 2:** Comparison of the ${\text{Re}}_{S}^{1/2} C_{fS}$ for different values of $\Omega$ and $\psi_{1} = \psi_{2} = \lambda = M = 0$.

$\Omega$	Rosca and Pop^51^	Afridi et al.^52^	Abbas et al.^53^	Ahmad et al.^54^	Dey et al.^55^	Present outcomes
_5_	1.15076	1.1576312	1.15763	1.157 630	1.157630	1.157623
_10_	1.07172	1.0734886	1.07349	1.073 490	1.073490	1.073493
_20_	1.03501	1.0356098	1.03561	–	–	1.035613
30	1.02315	1.0235310	1.02353	1.023530	1.0223	1.023532
40	1.01729	1.0175866	1.01759	–	–	1.017589
50	1.01380	1.0140492	1.01405	1.014050	–	1.014044
100	1.00687	1.0070384	1.00704	–	–	1.007036
200	1.00342	1.0035641	1.00356	1.003560	–	1.003559
1000	1.00068	1.0007993	1.00079	1.000790	–	1.000811

**Table 3:** Comparison of ${\text{Re}}_{S}^{ - 1/2} Nu_{S}$ for variations in $\Pr$ when $\Omega \to \infty$ and all other factors are zero.

$\Pr$	Khan and Pop^56^	Wang^57^	Gorla and Sidawi^58^	Devi and Devi^59^	Grubka and Bobba^60^	Gowda et al.^61^	Present results (bvp4c)
0.01	–	–	–	–	0.0099	0.00978	0.015664
0.2	0.1691	0.1691	0.1691	–	–	–	0.169088
0.7	0.4539	0.4539	0.5349	–	–	–	0.453917
0.72	–	–	–	–	0.4631	0.46273	0.463144
1.0	–	–	–	–	0.5820	0.58193	0.581976
2.0	0.9114	0.9114	0.9114	0.91135	–	–	0.911361
3.0	–	–	–	–	1.1652	1.16481	1.165252
6.13	–	–	–	1.75968	–	–	1.759698
7.0	1.8954	1.8954	1.8905	1.89540	–	–	1.895420
10	–	–	–	–	2.3080	2.30793	2.308034
20.0	3.3539	3.3539	3.3539	3.35390	–	–	3.353935
70.0	6.4622	6.4622	6.4622	–	–	–	6.462312
100.0	–	–	–	–	7.7657	7.76496	7.765856

## Discussion of results

**Figure 2:**
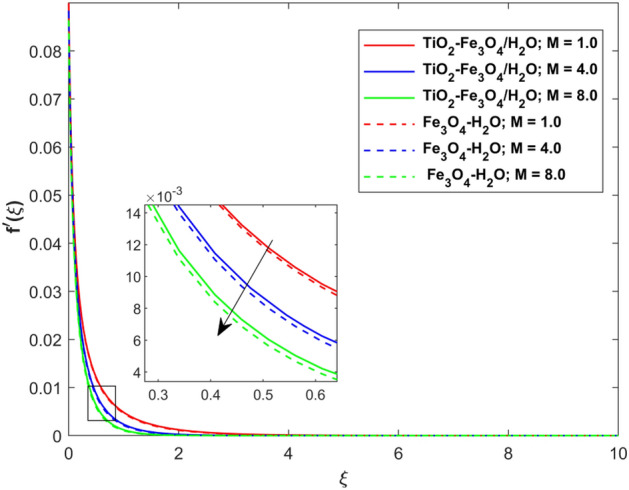
Impact of $M$ on $f^{\prime}\left( \xi \right)$.

**Figure 3:**
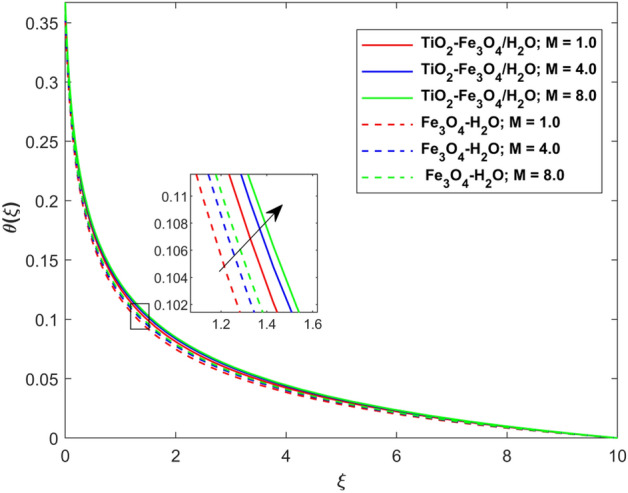
Impact of $M$ on $\theta \left( \xi \right)$.

**Figure 4:**
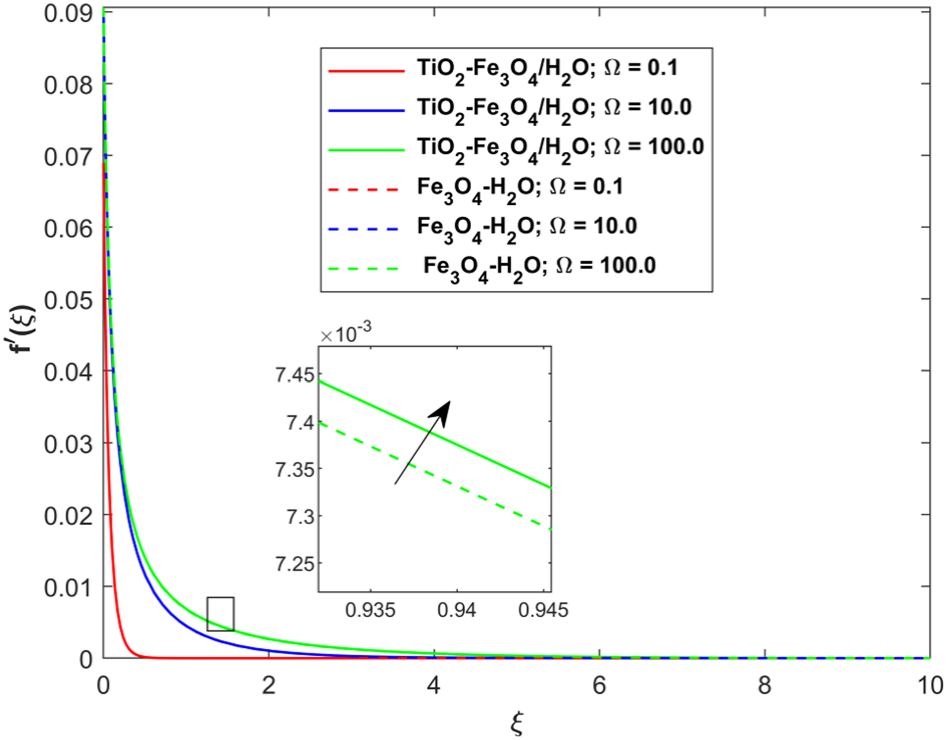
Impact of $\Omega$ on $f^{\prime}\left( \xi \right)$.

**Figure 5:**
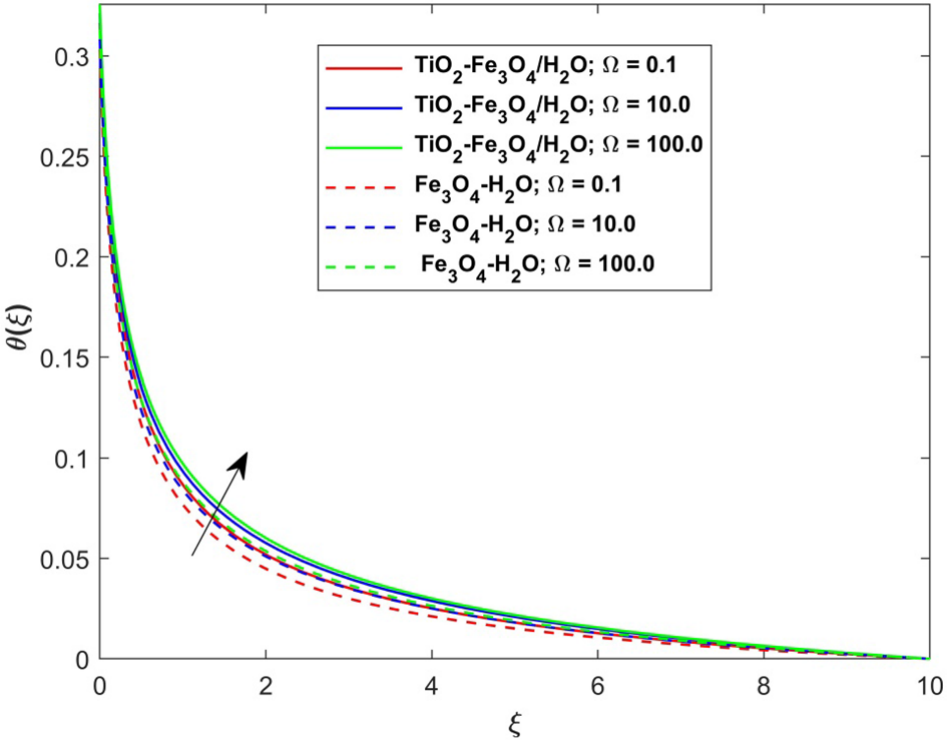
Impact of $\Omega$ on $\theta \left( \xi \right)$.

**Figure 6:**
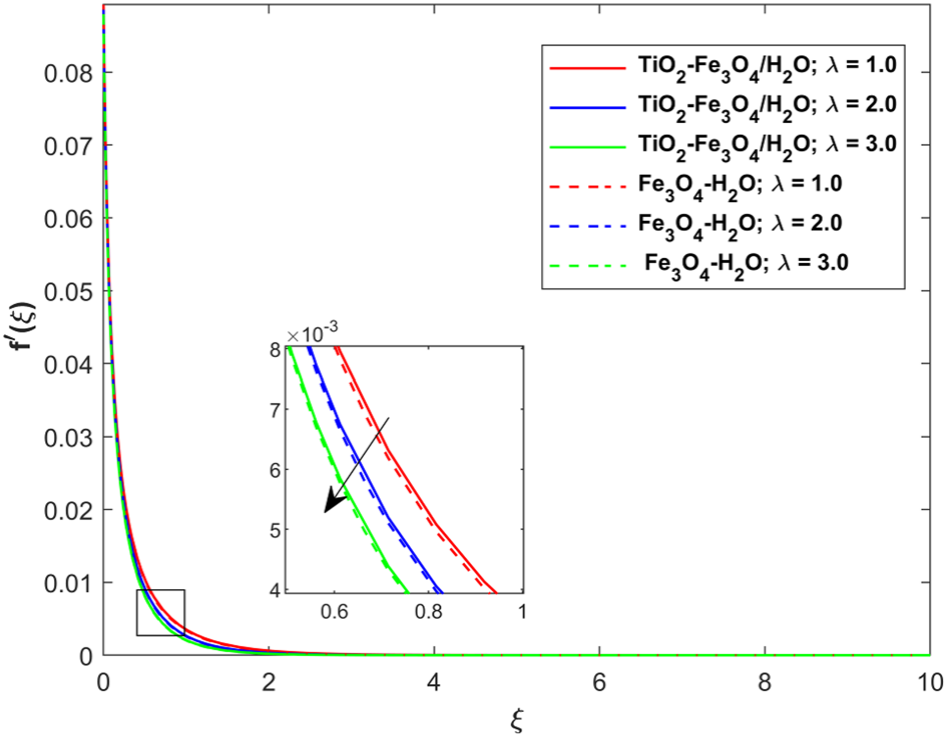
Impact of $\lambda$ on $f^{\prime}\left( \xi \right)$.

**Figure 7:**
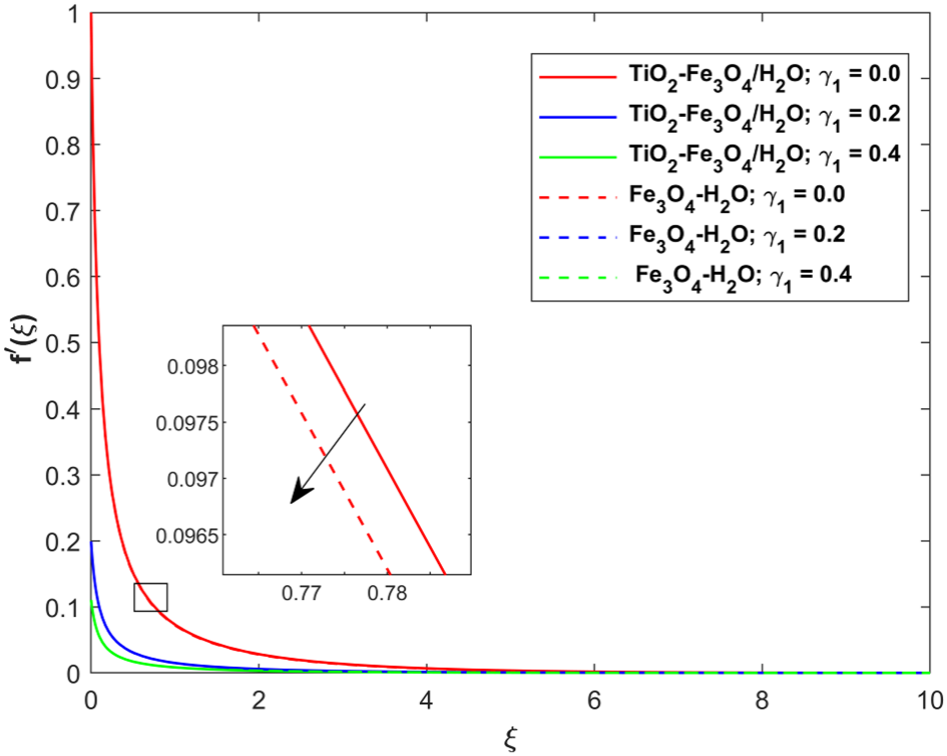
Impact of $\gamma_{1}$ on $f^{\prime}\left( \xi \right)$.

**Figure 8:**
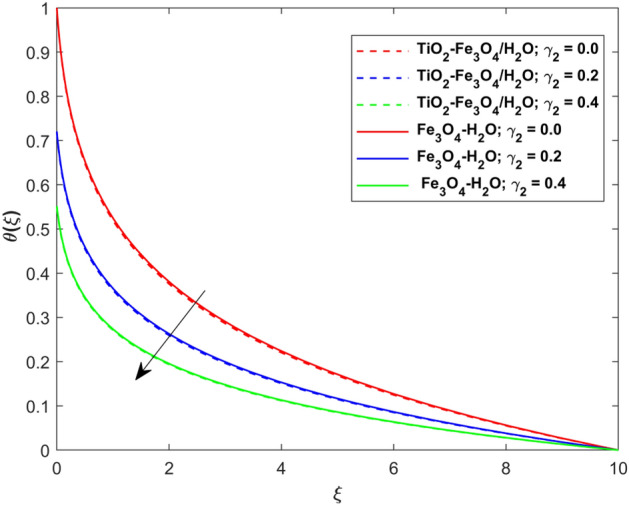
Impact of $\gamma_{2}$ on $\theta \left( \xi \right)$.

**Figure 9:**
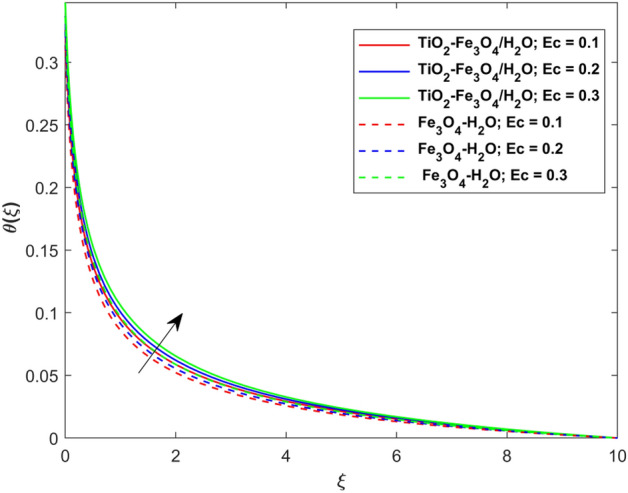
Impact of $Ec$ on $\theta \left( \xi \right)$.

**Figure 10:**
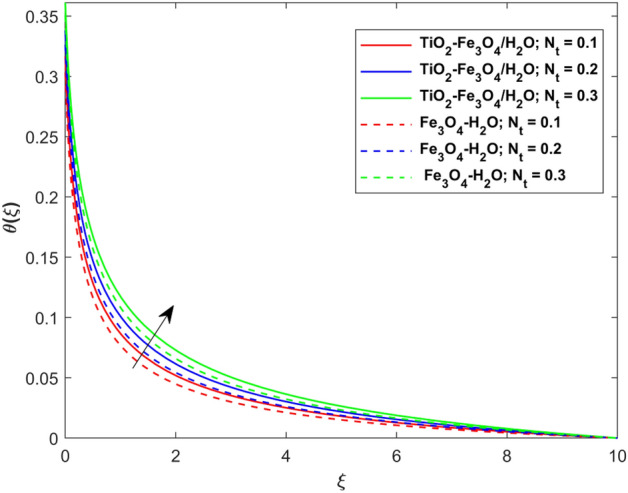
Impact of $N_{t}$ on $\theta \left( \xi \right)$.

**Figure 11:**
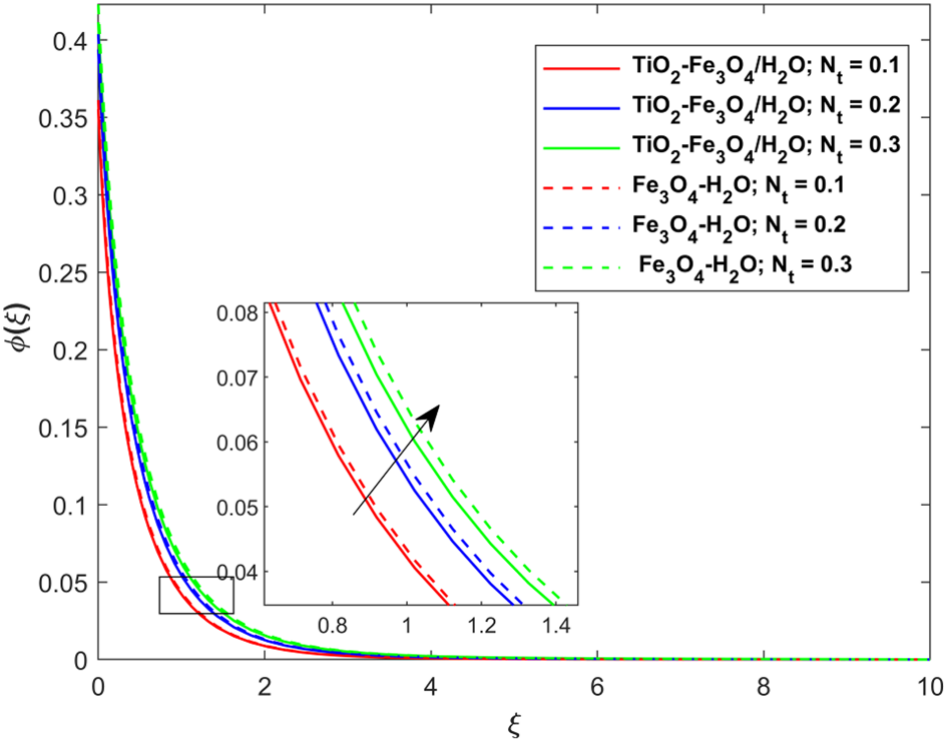
Impact of $N_{t}$ on $\phi \left( \xi \right)$.

**Figure 12:**
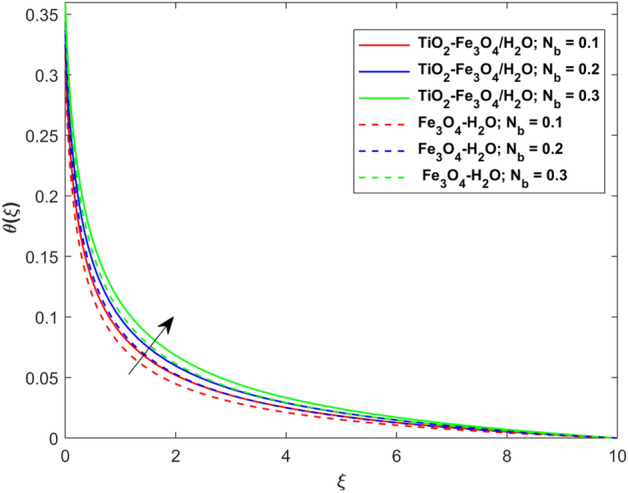
Impact of $N_{b}$ on $\theta \left( \xi \right)$.

**Figure 13:**
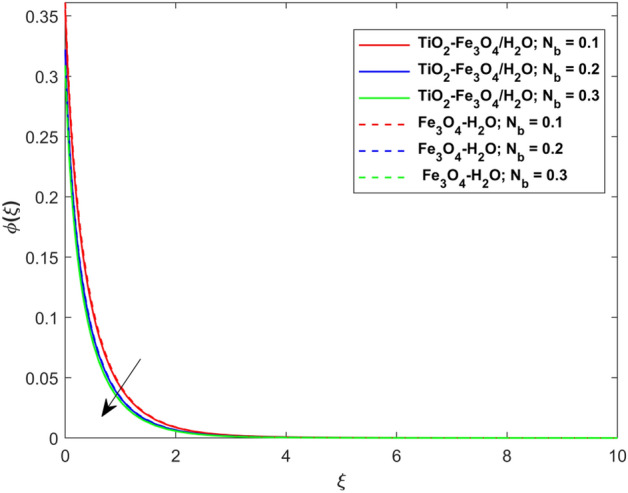
Impact of $N_{b}$ on $\phi \left( \xi \right)$.

**Figure 14:**
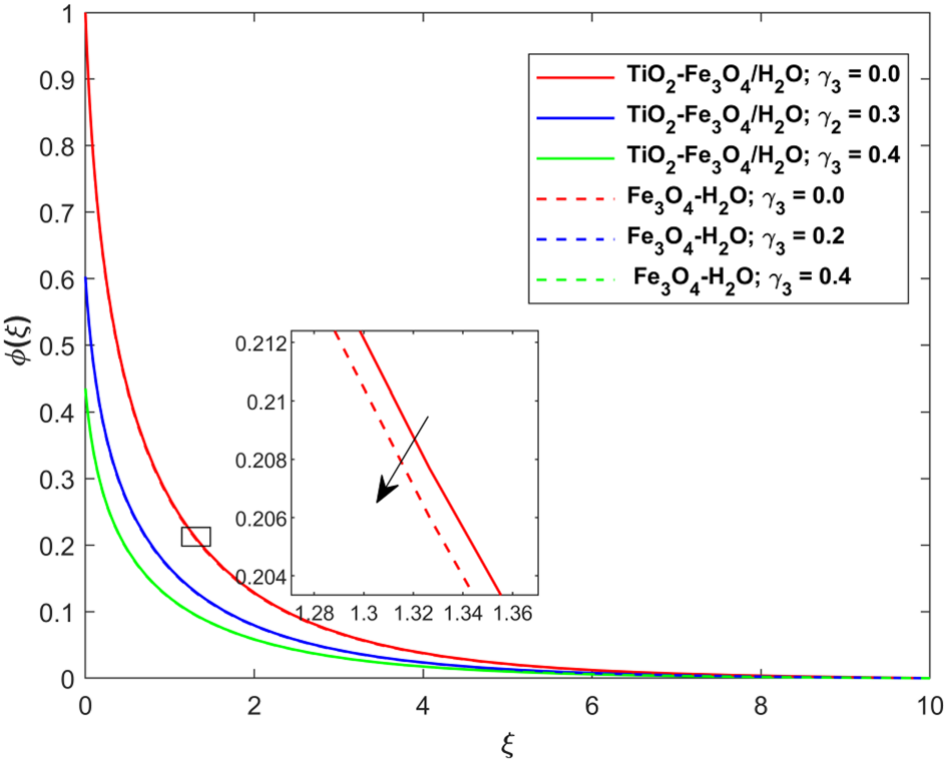
Impact of $\gamma_{3}$ on $\phi \left( \xi \right)$.

**Figure 15:**
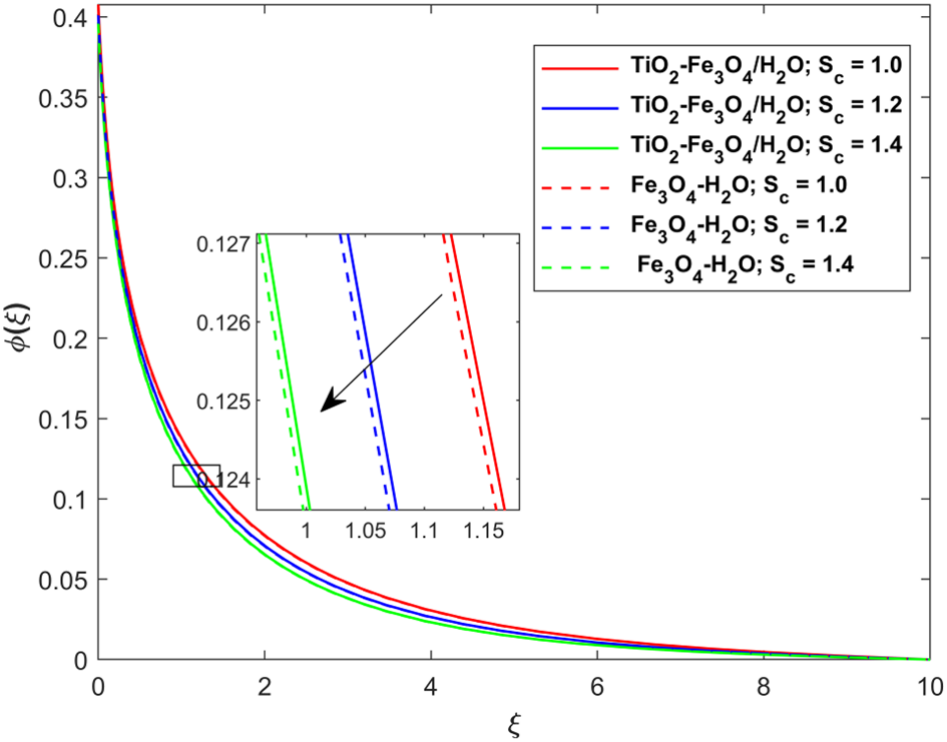
Impact of $S_{c}$ on $\phi \left( \xi \right)$.

**Figure 16:**
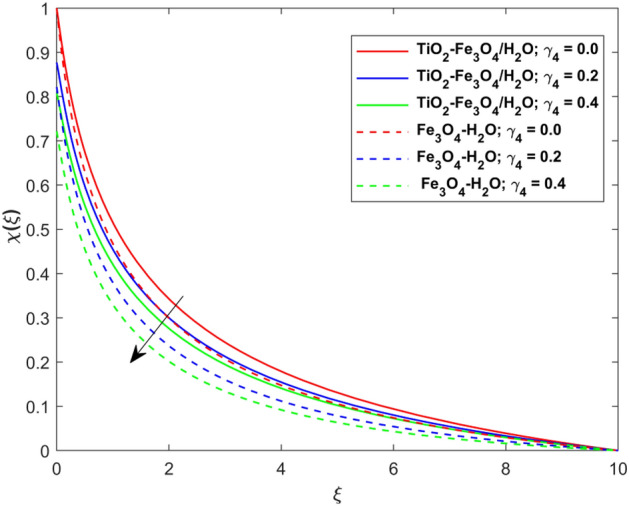
Impact of $\gamma_{4}$ on $\chi \left( \xi \right)$.

**Figure 17:**
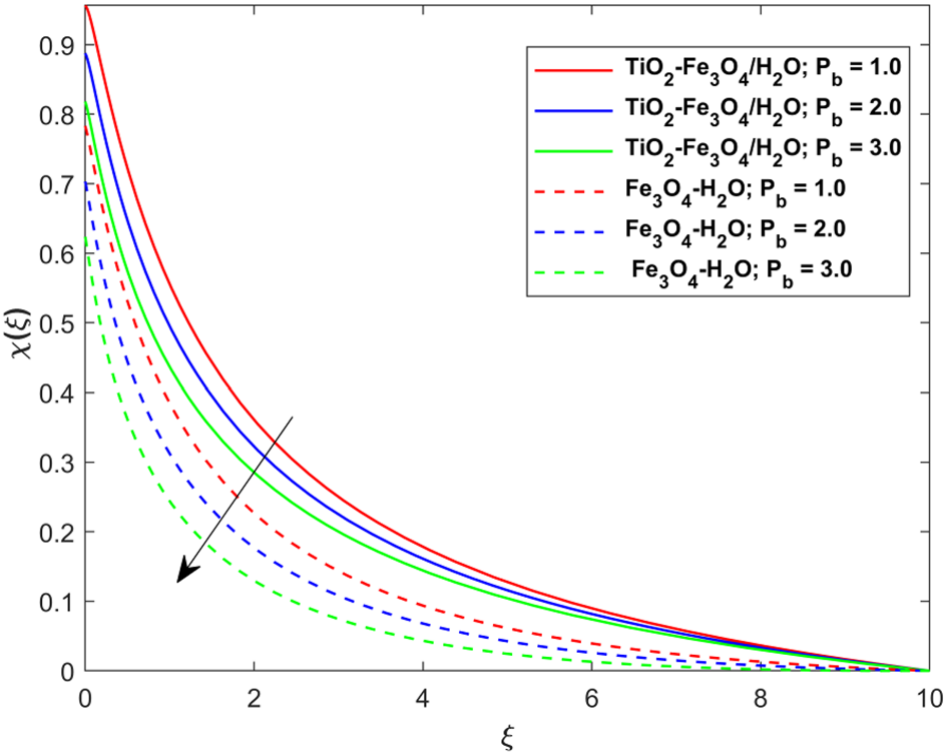
Impact of $P_{b}$ on $\chi \left( \xi \right)$.

This segment presents the impacts of the embedded factors on 2D flow of a water-based hybrid nanofluid flow comprising of gyrotactic microorganisms over a stretching surface using porous media. Figures 2, 3, 4, 5, 6, 7, 8, 9, 10, 11, 12, 13, 14, 15, 16 and 17 and Tables 4, 5, 6 and 7 are displayed. Figures 2 and 3 show the impact of magnetic factor ($M$) on velocity ($f^{\prime}\left( \xi \right)$) and thermal panels ($\theta \left( \xi \right)$), respectively. From these Figures, we observed that $f^{\prime}\left( \xi \right)$ reduces while $\theta \left( \xi \right)$ increases with higher $M$. Actually, Lorentz force is in direction relation to magnetic factor which means that with higher $M$, the Lorentz force (which is a resistive force to fluid motion) also increases. Thus, the increasing magnetic factor reduces the fluid particle motion, and causes reduction in the velocity profile. Conversely, with upsurge in $M$, the collision between the fluid particles increases which consequently increase the temperature profile as well. That’s why the higher temperature profile via magnetic factor is perceived. Figures 4 and 5 show variation in $f^{\prime}\left( \xi \right)$ and $\theta \left( \xi \right)$ via curvature factor ($\Omega$). By increasing $\Omega$, both the velocity and temperature distributions increase. Actually, the curvature term is in direct relation with radius of the curved factor. So with upsurge in curvature factor the curved surface became flat as discussed by Dawar et al.^62^. It is obvious that upsurge in curvature factor boost the velocity and temperature profiles due to the fact that both these profiles are having maximum growth at the flat surface than that of curve surface. Figure 6 shows the effects of porosity factor ($\lambda$) on $f^{\prime}\left( \xi \right)$. From this Fig. we see that the porosity factor acts against velocity distribution. That is the flow distribution reduces for higher values of porosity factor. The reason is that the permeability factor increase the friction force at the curved sheet which as a result lessens the fluid motion. Thus, $f^{\prime}\left( \xi \right)$ is the reducing function of $\lambda$. Figure 7 portrays the effects of velocity slip factor ($\gamma_{1}$) on $f^{\prime}\left( \xi \right)$. From this Figure we see that velocity panel is a reducing function of $\gamma_{1}$ as the higher values of $\gamma_{1}$ reduces $f^{\prime}\left( \xi \right)$. Figure 8 shows the influence of thermal slip factor ($\gamma_{2}$) on $\theta \left( \xi \right)$. From this Fig. we see that $\theta \left( \xi \right)$ is a reducing function of $\gamma_{2}$. The higher values of $\gamma_{2}$ reduces width of thermal layer at the boundary that results growth in $\theta \left( \xi \right)$. Figure 9 portrays the effects of Eckert number ($Ec$) on $\theta \left( \xi \right)$. It is clear that $\theta \left( \xi \right)$ is an escalating function of $Ec$. Upsurge in $Ec$ increases the inner heat which results in high energy curve. Thus, the increasing $Ec$ augments the $\theta \left( \xi \right)$. Figures 10 and 11 display the variation in ($\theta \left( \xi \right)$) and ($\phi \left( \xi \right)$) profiles via ($N_{t}$), respectively. From both Figures, we observed that $\theta \left( \xi \right)$ and $\phi \left( \xi \right)$ are the increasing functions of $N_{t}$. This is due to the fact that particles near to hot surfaces yield a thermophoretic force, which helps in particle decomposition beyond the liquid domain (on the curved surface) and results in an escalation in temperature and concentration layers thicknesses at boundary. Thus, both the concentration and temperature are the augmenting functions of $N_{t}$. Figures 12 and 13 show the variation in $\theta \left( \xi \right)$ and $\phi \left( \xi \right)$ via Brownian motion factor ($N_{b}$). From theses Figs. we see that $\theta \left( \xi \right)$ boots and $\phi \left( \xi \right)$ reduces via increasing $N_{b}$. Actually, with escalation in $N_{b}$ the width of thermal layer at the boundar magnified due to exchange of kinetic and thermal energies, hence escalation in $N_{b}$ causes a growth in $\theta \left( \xi \right)$. With larger amounts of $N_{b}$, the nanoparticle concentration profile slows down. Figure 14 depicts the effects of concentration slip factor ($\gamma_{3}$) on $\phi \left( \xi \right)$. From this Figure we see that concentration of nanoparticles distribution is a reducing function of $\gamma_{3}$. The higher values of $\gamma_{3}$ reduces concentration boundary layer thickness and as a result $\theta \left( \xi \right)$ reduces. Figure 15 shows the variation in $\phi \left( \xi \right)$ via Schmidt number ($S_{c}$). From this Fig. we see that upsurge in $S_{c}$ reduces $\phi \left( \xi \right)$. Actually, the higher values of $S_{c}$ reduces the Brownian diffusivity which causes the declination of mass curve. Thus, the higher values of $S_{c}$ reduces $\phi \left( \xi \right)$. Figure 16 shows the impact of microorganisms density slip factor ($\gamma_{4}$) on motile density distribution ($\chi \left( \xi \right)$). From this Fig. we see that the motile density distribution is a reducing function of $\gamma_{4}$. The higher values of $\gamma_{3}$ reduces motile density boundary layer thickness and as a result $\chi \left( \xi \right)$ reduces. Figure 17 shows the variation in microorganisms profile ($\chi \left( \xi \right)$) via bioconvection Peclet number ($P_{b}$). Here, it has noted that escalation in $P_{b}$ reduces $\chi \left( \xi \right)$. The generation of the rate of swimming mobile microbes by $P_{b}$ in the fluid causes a reduction in the width of microorganisms close to the surface. Table 4 portrays influence of $M$, $\lambda$ and $\gamma_{1}$ on surface drag force of the nano and hybrid nanoliquid. From this Table, we observed that upsurge in magnetic and porosity factors have increasing impact on ${\text{Re}}_{S}^{1/2} C_{fS}$ while the slip factor has reducing impact on it. Comparing the nano and hybrid cases, the greater impacts of these parameters are found on hybrid nanoliquid flow. Table 5 displays the impacts of $M$, $N_{t}$, $N_{b}$, $Ec$, $R_{d}$ and $\gamma_{2}$ on heat transfer rate of the nanoliquid and hybrid nanoliquid. From this Table, we see that the higher values of $M$, $N_{t}$, $N_{b}$, $Ec$ and $R_{d}$ factors have increasing impact on heat transfer rate while the higher values of $\gamma_{2}$ has reducing impact on it. It has perceived that greater impacts of these factors are found on hybrid nanofluid flow in comparison of nanofluid flow. Table 6 depicts the effects of $N_{t}$, $N_{b}$,$S_{c}$ and $\gamma_{3}$ on Sherwood number. From this Table, it has perceived that escalation in $N_{b}$ and $S_{c}$ factors have increasing impact on mass transfer rate while the higher values of $N_{t}$ and $\gamma_{3}$ has reducing impact on it. It has perceived that greater impacts of these factors are found on hybrid nanofluid flow in comparison of nanofluid flow. Table 7 portrays the impacts of $P_{b}$ and $\gamma_{4}$ on density number. From this Table, we see that the higher values of $P_{b}$ and $\gamma_{4}$ factors have reducing impact on density number. It has perceived that greater impacts of these factors are found on hybrid nanofluid flow in comparison of nanofluid flow.

**Table 4:** Numerical results of ${\text{Re}}_{S}^{1/2} C_{fS}$ for nanofluid and hybrid nanofluid.

$M$	$\lambda$	$\gamma_{1}$	${\text{Re}}_{S}^{1/2} C_{fS}$	
			Fe_3_O_4_–H_2_O	TiO_2_–Fe_3_O_4_/H_2_O
0.1			0.007663830	0.007997541
0.2			0.009861191	0.010192954
0.3			0.011994431	0.012332249
	0.1		0.007663830	0.007997541
	0.2		0.010122625	0.010731523
	0.3		0.012500262	0.013376189
		0.1	0.038644659	0.039629279
		0.2	0.019639768	0.020379759
		0.3	0.013033041	0.013515592

**Table 5:** Numerical results of ${\text{Re}}_{S}^{ - 1/2} {\text{Nu}}_{S}$ for nanofluid and hybrid nanofluid.

$M$	$N_{t}$	$N_{b}$	$Ec$	$R_{d}$	$\gamma_{2}$	${\text{Re}}_{S}^{ - 1/2} {\text{Nu}}_{S}$	
						Fe_3_O_4_–H_2_O	TiO_2_–Fe_3_O_4_/H_2_O
1						0.60963716	0.59734508
2						0.60991969	0.59759300
3						0.61014658	0.59779356
	0.1					0.52959009	0.53405039
	0.2					0.56295363	0.57125638
	0.3					0.59704042	0.60928442
		0.1				0.60928442	0.59704042
		0.2				0.64772447	0.63152795
		0.3				0.68599435	0.66600533
			0.1			0.59704042	0.60928442
			0.2			0.59706117	0.60930772
			0.3			0.59708192	0.60933102
				0.1		0.57450416	0.57476099
				0.2		0.59518931	0.59903332
				0.3		0.60928442	0.61704042
					0.1	0.95279244	0.96377161
					0.2	0.84190486	0.85493089
					0.3	0.74668786	0.7602522

**Table 6:** Numerical results of ${\text{Re}}_{S}^{ - 1/2} {\text{Sh}}_{S}$ for nanofluid and hybrid nanofluid.

$N_{t}$	$N_{b}$	$S_{c}$	$\gamma_{3}$	${\text{Re}}_{S}^{ - 1/2} {\text{Sh}}_{S}$	
				Fe_3_O_4_–H_2_O	TiO_2_–Fe_3_O_4_/H_2_O
0.1				0.28763452	0.28814559
0.2				0.26311552	0.26574013
0.3				0.22453709	0.22984022
	0.1			0.13128314	0.13821037
	0.2			0.19374403	0.19748745
	0.3			0.21596191	0.21837797
		1		0.11010277	0.12075775
		2		0.13029648	0.13742274
		3		0.13692688	0.14255733
			0.1	0.57222401	0.25757394
			0.2	0.37237090	0.37776014
			0.3	0.25757394	0.26381356

**Table 7:** Numerical results of ${\text{Re}}_{S}^{ - 1/2} {\text{Mn}}_{S}$ for nanofluid and hybrid nanofluid.

$P_{b}$	$\gamma_{4}$	${\text{Re}}_{S}^{ - 1/2} {\text{Mn}}_{S}$	
		Fe_3_O_4_–H_2_O	TiO_2_–Fe_3_O_4_/H_2_O
0.1		0.13128319	0.13821028
0.2		0.13128308	0.13821026
0.3		0.13128305	0.13821024
	0.1	0.13128314	0.13821037
	0.2	0.13128312	0.13821033
	0.3	0.13128310	0.13821031

## Conclusions

This article presents the 2D slip flow of hybrid nanofluid comprising of gyrotactic microorganisms on an elongating curved surface using porous media. The nanoparticles of *TiO*_*2*_ and *Fe*_*3*_*O*_*4*_ have dispersed in water for composition of hybrid nanofluid. Main equations of the problem have been converted to ODEs by using an appropriate set of variables. Solution of the present model is determined by bvp4c technique. The effects of various emerging factors on flow distributions have considered and explained. Additionally, the slips conditions are incorporated to analyze various flow distributions. By completing the present analysis, the following key points are concluded.

- The increasing magnetic factor diminishes the velocity curve while increases the temperature profile.
- Growth in curvature factor has magnified both temperature and velocity distributions.
- Upsurge in velocity, thermal, nanoparticle concentration, and microorganisms slip factors reduce the corresponding distributions.
- Thermal and nanoparticle concentration distributions are growing functions of thermophoresis factor. On the other hand, the temperature distribution increases with escalation in Brownian factor while the nanoparticle concentration distribution is the reducing function of that factor.
- The bioconvection Peclet number has reducing impact on the microorganisms’ distribution.
- The greater effects of embedded factors are found on hybrid nanofluid flow when compared to nanofluid flow.

## Data Availability

All data used in this manuscript have been presented within the article.
